# Conflict and control in error monitoring during sustained visual discrimination

**DOI:** 10.3389/fcogn.2026.1565885

**Published:** 2026-04-02

**Authors:** Taylor M. Curley, L. Jack Rhodes, Lorraine Borghetti, Megan B. Morris

**Affiliations:** 1 Air Force Research Laboratory, Wright-Patterson Air Force Base, Dayton, OH, United States; 2 BAE Systems at Air Force Research Laboratory, Wright-Patterson Air Force Base, Dayton, OH, United States

**Keywords:** attention, control, discrimination, effort, fatigue, vigilance

## Abstract

Increases in response errors across time-on-task are ubiquitous in vigilance research, particularly for tasks that require visual operations. An important aspect of vigilance tasks is response monitoring, particularly when a prepotent response must be inhibited. Neural indices of performance monitoring, such as the error-related negativity (ERN), provide strong evidence for changes in response conflict across time during vigilance tasks; however, it is unclear whether the allocation of additional cognitive resources (i.e., working memory) during such tasks leads to differences in response errors and performance monitoring. To examine this relationship, we employed a visual discrimination task in which participants must compare pairs of lines either to each other (simultaneous discrimination) or to a template held in working memory (successive discrimination) and decide whether to withhold or provide a response across 4 blocks of trials. We predicted lower ERN amplitudes and greater false alarm rates during visual discrimination trials that employ working memory (successive discrimination) compared to those that do not (simultaneous discrimination). While ERN peak amplitude was significantly greater during simultaneous compared to successive discrimination for incorrect responses, participants showed significantly higher false alarm rates during simultaneous discrimination, contrary to our hypotheses. To further investigate these results, we developed and examined outputs from computational models of the two experimental tasks based on a parallel-distributed processing account of cognitive control in which working memory (during successive discrimination), response conflict, and effortful control modulate continuous task performance. Task simulations suggest that pre-stimulus processing of a working memory template during successive discrimination helps to lower false alarms and alleviate post-stimulus error monitoring. These results are interpreted in the context of resource allocation and compensatory control with respect to the vigilance decrement.

## 1 Introduction

Vigilance, the ability to sustain attention over time, and the vigilance decrement, the waning of vigilant performance over time, have been important areas in human performance research given their ubiquity in day-to-day (e.g., driving) and workplace (e.g., quality control) activities (Neigel et al., 2020). Despite decades of research focused on the vigilance decrement, advancements in theory are still being revealed by researchers (e.g., Thomson et al., 2015). These theoretical advancements can help practitioners build more accurate and predictive models to aid in combating the vigilance decrement by informing the design of interfaces and human-machine teaming technologies.

A difficult aspect of examining theories of sustained attention is the wide range of complementary cognitive processes that can modulate the vigilance decrement. For example, there are significant differences in response patterns attributable to simple reactions to stimuli (“arousal vigilance”) and those attributable to the detection of infrequent signals (“executive vigilance”; Luna et al., 2018, 2022). One potential avenue for resolving theoretical arguments is to bridge traditional accounts of the vigilance decrement with patterns extracted from neurocognitive methods, such as brain imaging and electroencephalography. To this end, the present article examines the relationship between sustained attention and working memory—an executive process that has been identified as a strong covariate of vigilance (Caggiano and Parasuraman, 2004; Helton and Russell, 2011)—and its implications for theories of the vigilance decrement using a combination of experimental, neurocognitive, and modeling methods. We expect that differences in error rates between tasks that do and do not engage working memory, along with neural indices of post-response error monitoring in the frontal cortex, will reflect an integrated balance between cognitive resource depletion and compensatory effort across time-on-task.

### 1.1 Vigilance decrement frameworks

Two main theoretical camps have dominated the cognitive effort literature. Overload theories argue that vigilance tasks are inherently demanding, resulting in reduced performance over time. Specifically, cognitive resource theory posits a limited cognitive resource pool that is diminished over time while completing a demanding task (Neigel et al., 2020). Given the continuous nature of vigilance tasks, these resources cannot be replenished in time, leading to a vigilance decrement (Warm et al., 2018). Research suggests that this is particularly true for working memory, which is hypothesized to be an important resource underlying vigilant attention. For example, Caggiano and Parasuraman (2004) demonstrated greater time-on-task decreases in response accuracy when participants were required to use spatial working memory compared to non-spatial working memory. Similarly, a set of experiments by Helton and Russell (2011) found significant performance decrements in vigilance tasks that require the use of both spatial and verbal working memory compared to those that only require the use of one. These results suggest that there are limited resources required for the short-term storage of response-related information and the authors argue in favor of a multiple resource/overload account of vigilance.

On the other hand, underload theories, such as mindlessness theory and mind wandering theory, argue that vigilance tasks are so undemanding that they lead to automatic responding and attentional lapses, likely shifting attention from the task to task-unrelated thoughts over time (Manly et al., 1999; Robertson et al., 1997; Smallwood and Schooler, 2006). More recent theoretical perspectives have focused on integrations of different theoretical accounts to explain gaps and divergent findings. For example, Thomson et al. (2015)'s resource control theory integrates cognitive resource theory and mind wandering theory, suggesting that cognitively demanding vigilance tasks strain central attentional control over time to where attentional resources are shifted to self-generated thought to ease strain.

Traditional accounts of vigilance are also informed by theories that provide an integrated framework for the neural and behavioral components that underlie cognitively-demanding tasks. Sustained attention, for example, has been linked to multiple brain-stem-thalamo-cortical pathways (Oken et al., 2006) that regulate sleep-wake states (Dijk and Czeisler, 1995), up-regulation and sensitivity to performance-enhancing supplements (e.g., caffeine; Brunyé et al., 2010; Cappelletti et al., 2015), the secretion of neuromodulators that are key to the regulation of attention and arousal (Sara, 2009), and many others. In particular, researchers have focused on cortical activity localized in the fronto-central region of the brain that reliably correlates with a large range of cognitive tasks related to effort, control, and motivation (Badre and Nee, 2018; Friedman and Robbins, 2022; Shenhav et al., 2017).

### 1.2 Error monitoring and control in the ACC

While there are several frontal regions that correlate with cognitive control, such as the medial and lateral prefrontal cortex (PFC; Koechlin et al., 2003; Kouneiher et al., 2009; Ridderinkhof et al., 2004), the anterior cingulate cortex (ACC) has been particularly instrumental in the formation of neurocognitive theories of motivation and control. Beginning with early observations linking the region to executive functioning (e.g., Pardo et al., 1990; Paus et al., 1993; Vogt et al., 1992), neurocognitive assessments of the ACC have localized “cognitive” and “affective” components to the dorsal and ventral areas of the anterior executive region, respectively (Bush et al., 2000; Devinsky et al., 1995), and linked overall ACC functioning to a wide range of complex behaviors, such as social evaluation (Apps et al., 2016; Cunningham and Zelazo, 2007; Rotge et al., 2015), emotional awareness (Duncan and Barrett, 2007; Lane et al., 1998), and reward-based decision-making (Bush et al., 2002; Kennerley et al., 2006).

The most consistent and widely-researched neural signal related to the ACC is the temporally precise error-related negativity (ERN) event-related potential (ERP)—a negative electro-cortical inflection that peaks within 100 ms, the size of which is dependent upon correct (smaller amplitude) or erroneous (larger amplitude) responses during choice reaction-time tasks (Falkenstein et al., 1991; Gehring et al., 1993, 2012; Hajcak et al., 2005). Initial accounts of the ERN were somewhat limited in scope, postulating that the signal reflects general error processing (Coles et al., 2001; Falkenstein et al., 1991) and negative reinforcement (Holroyd and Coles, 2002), but more recent theories have revised these viewpoints to be more expansive accounts of cognitive control, particularly with respect to high-level performance monitoring (e.g., Botvinick et al., 2001; Yeung et al., 2004). Currently, theories of ACC functioning and the role of the ERN provide a comprehensive view of a system that integrates disparate pieces of information, such as the cost of cognitive control (Shenhav et al., 2013) and negative arousal following critical trials (Inzlicht and Al-Khindi, 2012; Inzlicht et al., 2015) into a cortical signal following a response. An open question in this line of research, however, is how ERN correlates of mental fatigue and response error rates across time-on-task (e.g., Boksem et al., 2006; Xiao et al., 2015) relate to these neurocognitive models of effort and control.

### 1.3 Hybrid framework of fatigue

A new account of time-on-task fatigue has recently been proposed by Borghetti et al. (2024): the Hybrid Framework of Fatigue. The hybrid framework unites the explanatory advantage of a renowned cognitive-energetic theory of motivation—the compensatory control model, or CCM (Hockey, 1997, 2011)—with the rigorous formalisms of recent computational models of fatigue, or CMFs (Curley et al., 2024; Gunzelmann et al., 2009; Veksler and Gunzelmann, 2018), and high-fidelity advantages of neurophysiological data. Here, trade-offs between competing motivational states (effortful on-task motivational control vs. a low-effort unstressed off-task state) are computationally instantiated as microlapses, defined as brief transitory respites from aversive centrally-controlled effort. The resulting interplay reflects a dynamic (i.e., fluctuating over time due to competing motivations) and compensatory (i.e., boosts in effort in response to fatigue effects) system vulnerable to the build-up of time-on-task effects (an increasing desire to remain in an off-task state). For example, high gamma (70–100 Hz) neural oscillatory activity was parameterized to index effortful motivational control in a set of computational models (Borghetti et al., 2024; Curley et al., 2024). While assumed to moderate trade-space activity, the hybrid framework does not specify a neural analog for determining *when* to transition between on- and off-task states, a mechanism referred to as the “effort monitor”. The current work seeks to identify the temporal and neurocognitive processes to address the gap.

### 1.4 Current effort

The current work investigates a plausible neural instantiation of an effort monitor mechanism originally posited in Hockey's CCM (Hockey, 1997) and assumed in the hybrid framework (Borghetti et al., 2024). According to the CCM, effort for well-learned skills aligned with task-relevant performance goals occurs without substantial costs and, consequently, the individual avoids distress. As an individual's performance degrades and falls below a threshold for task goals, an “effort monitor” mechanism determines whether to switch to a controlled on-task state requiring more effort to meet the performance threshold or reduce effort (and therefore, performance) to maintain well-being. Both strategies allay differences between task goals and the current state but differentially advantage task performance vs. biological comfort.

The ERN can potentially play the role as an effort monitor during demanding tasks (LoTemplio et al., 2023). The ERN correlates with reward predictions and response switching (Yasuda et al., 2004) and affective reward processing mediated by the ACC, a frontal region linked to conflict monitoring and resolution (Inzlicht et al., 2015). Additionally, the ERN and ACC are also associated with complex behaviors that evaluate the expected value of allocating effort based on performance costs and payoffs (Inzlicht et al., 2018; Shenhav et al., 2013, 2017).

To examine differences in vigilant effort, we first employed two versions of a simple discrimination task: One that requires a template of a target stimulus to be held in working memory (i.e., *successive* discrimination) vs. one that does not (i.e., *simultaneous* discrimination). Given prior research on working memory and the vigilance decrement (Helton and Russell, 2011; Warm and Dember, 1998), we expect that the task that requires the engagement of working memory will result in significantly larger performance decrements across time-on-task, particularly as they relate to false alarm rates. This would reflect prior research suggesting that successive discrimination tasks are more mentally demanding compared to simultaneous discrimination tasks (e.g., Caggiano and Parasuraman, 2004) and extend reaction time (RT) due to these additional demands (Gartenberg et al., 2018). To compliment the task, we also used electroencephalography (EEG) to examine frontal ERN during response errors. We expect that ERN amplitudes for both tasks will significantly increase across time-on-task, reflecting prior research suggesting that ERN amplitudes increase during prolonged Go/No-go tasks (Clayson et al., 2025). Similarly, we expect to find higher ERN amplitudes during successive discrimination, reflecting greater working memory engagement and, in turn, greater levels of mental effort.

## 2 Experimental methods

### 2.1 Participants

Thirty community-dwelling adult volunteers (*N*_*female*_ = 18; *Range*_*age*_ = 18-26; *M*_*age*_ = 21.17; *SD*_*age*_ = 2.23) recruited through the University of Dayton Research Institute (UDRI) participated in two separate 2-h sessions, each occurring on different days. During each session, participants were fitted with EEG caps and asked to participate in one of the two stimulus conditions (simultaneous vs. successive discrimination). The order of these within-subject conditions was counterbalanced for each participant. The study was approved by Institutional Review Boards at both UDRI and the Air Force Research Laboratory (AFRL), and all individuals were compensated for their participation in the study.

### 2.2 Materials and apparatus

**Figure 1:**
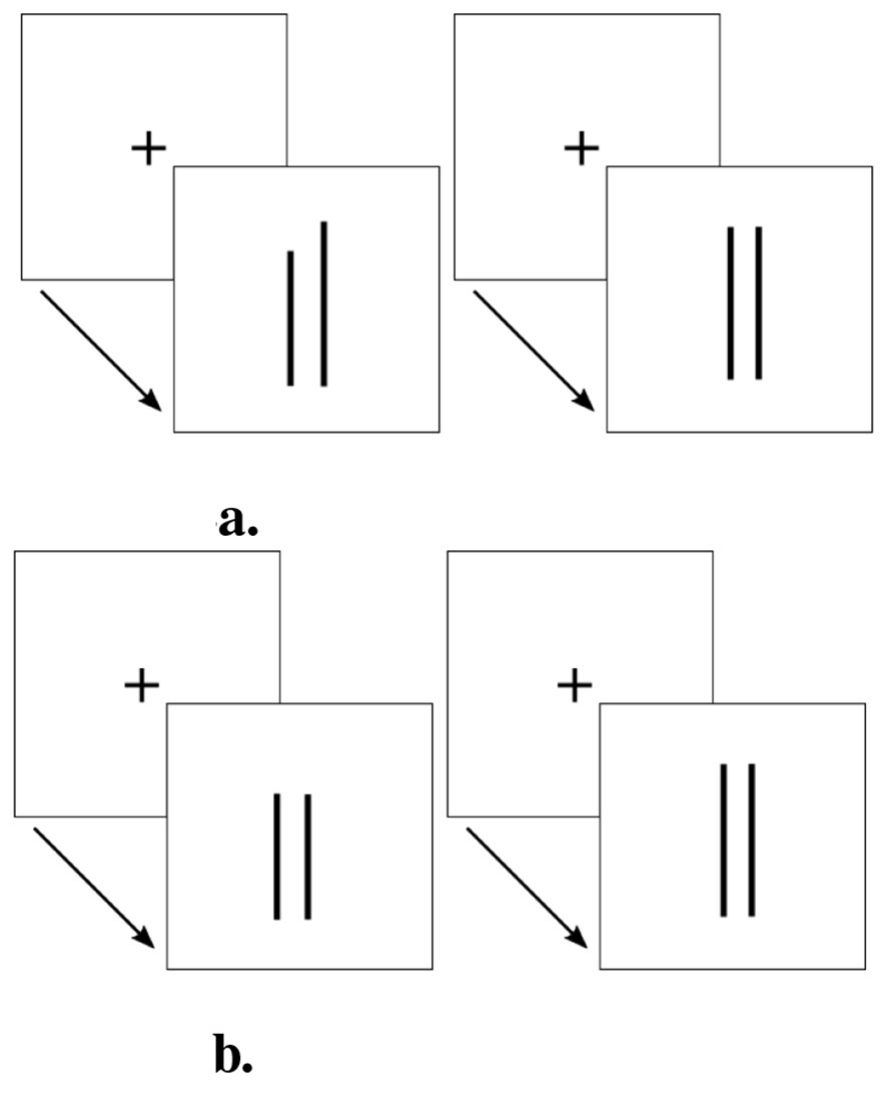
Examples of stimuli presented during the study. In the simultaneous task **(a)**, the stimuli could either be 2 lines of different (left) or similar (right) lengths. In the successive task **(b)**, the stimuli could either be 2 “short” (left) or 2 “long” (right) lines. Note that these figures are presented for readability; the actual stimuli presented during the task were white lines against black backgrounds.

Individuals were seated at desks containing Dell (64 bit, Intel Xeon 3.10 GHz processor, 16 GB RAM, Windows 7 OS) desktop computers running MATLAB (version R2023b), each with Dell UltraSharp U3014t monitors set to a 60 Hz refresh rate and luminance of 300 cd/m^2^, in a dimly-lit room to reduce glare and other environmental artifacts. Based on prior RT norming data (e.g., Tang et al., 2025), the devices were judged to have measurement latencies of no more than 50 ms. Participants were asked to engage in a simple visual discrimination task in which pairs of vertically-oriented lines are shown on the screen (Figure 1). They were instructed to provide a keyboard response as quickly and accurately as possible only when they see a critical stimulus configuration. Both presented lines were 0.75 mm in width and horizontally separated by 7 mm.

During each trial, these lines were presented centrally on a computer screen approximately 60 cm from the participant for 150 ms, and the time in between trials—or the inter-stimulus interval (ISI)—was randomly-chosen from a uniform distribution bounded between 1300 and 1700 ms. Other differences in presentation were governed by experimental conditions, which are described in more detail below.

There were three within-subjects manipulations: discrimination task type (*simultaneous* vs. *successive*), event type (*critical* vs. *non-critical*), and session (*Day 1* vs. *Day 2*). To prevent stimulus and practice effects, participants engaged in 4 counterbalanced blocks of trials, each with different task and event configurations:

1. Simultaneous task with matching lines as the critical stimulus,
2. Simultaneous task with lines of different lengths as the critical stimulus,
3. Successive task with “long” lines as the critical stimulus, and
4. Successive task with “short” lines as the critical stimulus.

Each session consisted of 100 practice trials followed by 4 blocks of 100 test trials, with 10% of all trials containing a critical stimulus. In all, participants completed 100 practice trials and 1,600 test trials across 4 counterbalanced conditions.

#### 2.2.1 Task type

**Figure 2:**
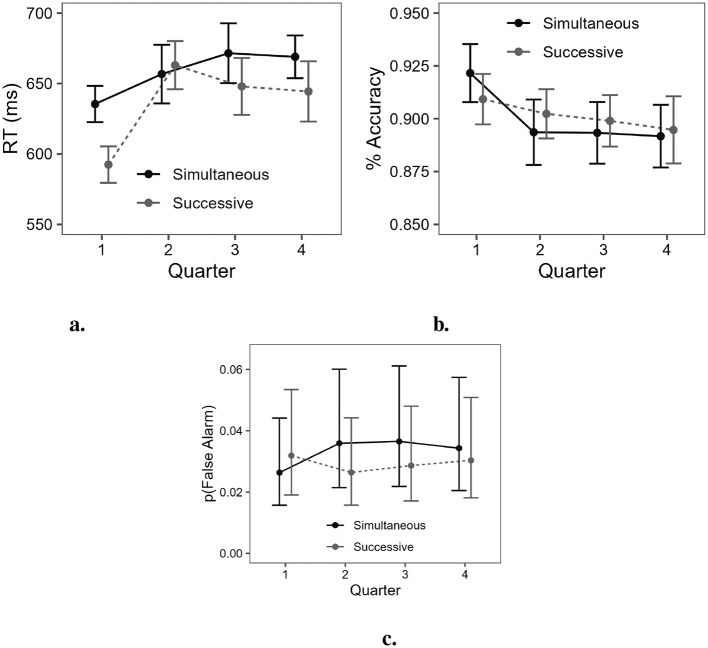
Overall reaction times **(a)**, accuracy rates **(b)**, and false alarm rates **(c)** from the simultaneous (solid lines) and successive (dotted lines) discrimination tasks.

The critical difference between successive and simultaneous task types involves the use of working memory. For simultaneous task trials, each presented line is either 16.7 mm (“short”) or 18 mm (“long”) in length. During these trials, participants are instructed to respond either if the presented lines are the *same* height or *different* heights; thus, all that is needed to make a correct discrimination is a simple visual comparison of the two lines matched against the response criteria (e.g., Figure 2a). In the *successive* task, however, both of the presented lines always have the same height (e.g., Figure 2b). During these trials, participants are asked to respond only to lines that are both “long” (18 mm) or “short” (14.6 mm) compared to the rest. Successful discrimination, then, relies on comparison of the currently-presented stimuli to a template of the target stimulus held in working memory. We used data from pilot testing to ensure that the differences between the long and short stimuli in both experimental conditions led to objective discriminability (*d*′) of 2.0, ensuring equal perceptual difficulty in both tasks (c.f., Parasuraman and Mouloua, 1987).

#### 2.2.2 Event type

Task events were dependent upon whether the presented stimuli matched the target configuration (i.e., a *critical* event) or not (i.e., a *non-critical* event). Additionally, we labeled different types of responses given within event types. If a participant correctly responded during a critical trial, the event was coded as a “hit”. Similarly, if a participant correctly withheld a response during a non-critical trial, the event was coded as a “correct rejection”. Trials in which responses were given when the presented stimuli did not match the target configuration, however, were labeled as “false alarms”, and trials without a valid response when the presented stimuli matched the target were labeled as “misses”. Given the scope of the current paper, we focused our analyses on hits and false alarm trials while excluding misses and other rare response errors, such as responses given faster than 200 ms, i.e., “false starts”.

### 2.3 Electrophysiology

EEG data collection and preprocessing procedures are fully described in Morris et al. (2022). Briefly, we applied 64 electrodes following the international 10–20 system, with additional mastoid sensors acting as the reference. Data were recorded at 512 Hz and bandpass filtered between 0.1 and 70 Hz, with an additional notch filter between 59.5 and 60.5 Hz to address any potential line noise in *EEGLAB* (Delorme and Makeig, 2004). Eye-blinks and other myogenic artifacts were removed with independent component analysis decomposition (Ablin et al., 2018) and custom in-house MATLAB scripts. For each participant, data for each trial with a false alarm were epoched into 2,700 ms segments including a 200 ms baseline period prior to stimulus onset, and baseline corrected from −200 to 0 ms. Next, EEG data was time locked to the response, retaining 500 ms prior to and 1,500 ms after the response. EEG data were organized into separate bins for each Quarter of the experiment. Based on literature review (c.f., Gehring et al., 2012; Yeung et al., 2004) and visual inspection of time-segmented topological plots, we selected data from sensors AFz, Fz, and FCz (midline fronto-central) and examined the ERN between 0 and 150 ms after response. Our aggregate behavioral and EEG analyses consider only false alarm trials for which EEG data is available (that is, omitting trials for which EEG data was rejected in preprocessing) during Simultaneous (*N*_*Q*1_ = 407, *N*_*Q*2_ = 404, *N*_*Q*3_ = 403, *N*_*Q*4_ = 395) and Successive (*N*_*Q*1_ = 493, *N*_*Q*2_ = 489, *N*_*Q*3_ = 483, *N*_*Q*4_ = 481) discrimination.

### 2.4 Statistical analyses

False alarm RTs were calculated as the time from stimulus onset to participant response on trials with false alarms. ERN peak amplitude was assayed through determining the lowest amplitude between 0 and 150 ms post-response, and ERN peak latency is the time (ms) of the ERN amplitude minimum within that same time window. Statistically, these factors were assessed separately using the desired feature as the dependent variable, and Task and Quarter as independent variables. All ANOVA models were evaluated using Type III Sums of Squares. Where appropriate, *post-hoc* *t*-test comparisons are reported after applying a Bonferroni correction. All statistical analyses were conducted in R (version 4.4.1; R Core Team, 2022) using the *lme*4 (version 1.1-37) Bates et al. (2015), *rstatix* (version 0.7.2) Kassambara (2023), and *stats* (version 4.4.1) packages, as well as *EEGLAB* (Delorme and Makeig, 2004) in MATLAB (version R2023b).

## 3 Experimental results

Here, we present the results of the experiment relating to response, latency, and ERN patterns, particularly as they relate to false alarm errors. Other metrics, such as end-spurt ERP patterns, have been reported elsewhere (c.f., Morris et al., 2022).

### 3.1 Behavioral

#### 3.1.1 Overall response latency

We conducted a 2 (Task: Simultaneous vs. Successive) x 4 (Quarter: 4 blocks of 400 trials) within-subjects ANOVA, with Greenhouse-Geisser corrections on degrees of freedom where assumptions of sphericity were violated (Figure 2a). For RTs, there was only a significant main effect of Quarter, *F*(1.45,33.37) = 7.01, *p* < 0.01, reflecting a significant increase between Quarter 1, *M* = 613.52, *SE* = 9.48, and Quarter 2, *M* = 659.48, *SE* = 16.15, *t* = 3.21, *p* < 0.01. Additionally, RTs significantly decreased between Quarters 3, *M* = 659.48, *SE* = 14.61, and 4, *M* = 653.41, *SE* = 13.17, *t* = 2.99, *p* < 0.01.

#### 3.1.2 Accuracy

A similar pattern emerged for accuracy (Figure 2b), where there was also main effect of Quarter, *F*(1.65,37.86) = 2.71, *p*= 0.05, though with marginal significance. A partial interaction contrast indicates that accuracy was significantly higher for Quarter 1, *M* = 0.91, *SE* = 0.01, compared to all other quarters, *M*s = 0.89, *SE*s = 0.01, χ^2^(1) = 6473.10. *p* < 0.05. There were no significant main effects of Condition, nor were there any significant interactions between Task and Quarter, *ps*> 0.05.

#### 3.1.3 False alarm rates

We further probed response patterns by examining only false alarm responses (Figure 2c). We submitted trial-level false alarm responses to a 2 (Task) x 4 (Quarter) generalized linear model with a logit link and random intercepts for each participant, using Quarter 1 and the Simultaneous condition as the comparison groups. The resulting model provides a good fit to the data, Pseudo-*R*^2^ = 0.37, where most of the information in the model is attributable to the grouping variable, *ICC* = 0.37. Estimates for the interaction between Task and Quarter are all significant, *p*s < 0.05, reflecting large qualitative differences in false alarm rates across blocks and conditions. Specifically, for Simultaneous discrimination trials, paired comparisons indicate that false alarm rates during Quarter 1 (*M* = 0.026, *SE* = 0.006) were significantly lower than those for Quarter 2 (*M* = 0.036, *SE* = 0.007, *z* = −5.32, *p* < 0.01), Quarter 3 (*M* = 0.037, *SE* = 0.006, *z* = −5.64, *p* < 0.01), and Quarter 4 (*M* = 0.034, *SE* = 0.008, *z* = −4.51, *p* < 0.05), although no other comparisons were significant, *p*s > 0.05. In contrast, for Successive discrimination trials, false alarm rates during Quarter 1 (*M* = 0.032, *SE* = 0.007) were significantly greater than those during Quarter 2 (*M* = 0.026, *SE* = 0.006, *z* = 3.10, *p* = 0.01), but not compared to Quarters 3 (*M* = 0.028, *SE* = 0.006, *z* = 1.78, *p* = 0.29) and 4 (*M* = 0.030, *SE* = 0.006, *z* = 0.84, *p* = 0.84), and no other comparisons were significant, *p*s > 0.05. Main effects estimates indicate significant differences in false alarm rates between conditions, β = 0.19, *SE* = 0.06, *z* = 3.14, *p* < 0.01, reflecting significantly higher false alarm rates in the Simultaneous task, *M* = 0.032, *SE* = 0.008, compared to the Successive task, *M* = 0.028, *SE* = 0.007. Similarly, significant omnibus model estimates for Quarter 2, β = 0.31, *SE* = 0.06, *z* = 5.32, *p* < 0.01, Quarter 3, β = 0.33, *SE* = 0.06, *z* = 5.64, *p* < 0.01, and Quarter 4, β = 0.26, *SE* = 0.06, *z* = 4.51, *p* < 0.01, reflect a significant difference in false alarm rates between Quarter 1 (*M* = 0.028, *SE* = 0.007) and Quarter 3 (*M* = 0.031, *SE* = 0.008, *z* = −2.63, *p* = 0.04) and a difference between Quarters 1 and 4 (*M* = 0.031, *SE* = 0.008, *z* = −2.56, *p* = 0.05) of marginal significance. The comparison between Quarter 1 and Quarter 2 (*M* = 0.030, *SE* = 0.008) was not significant, nor were any other paired tests, *ps*> 0.05.

#### 3.1.4 False alarm latency

**Figure 3:**
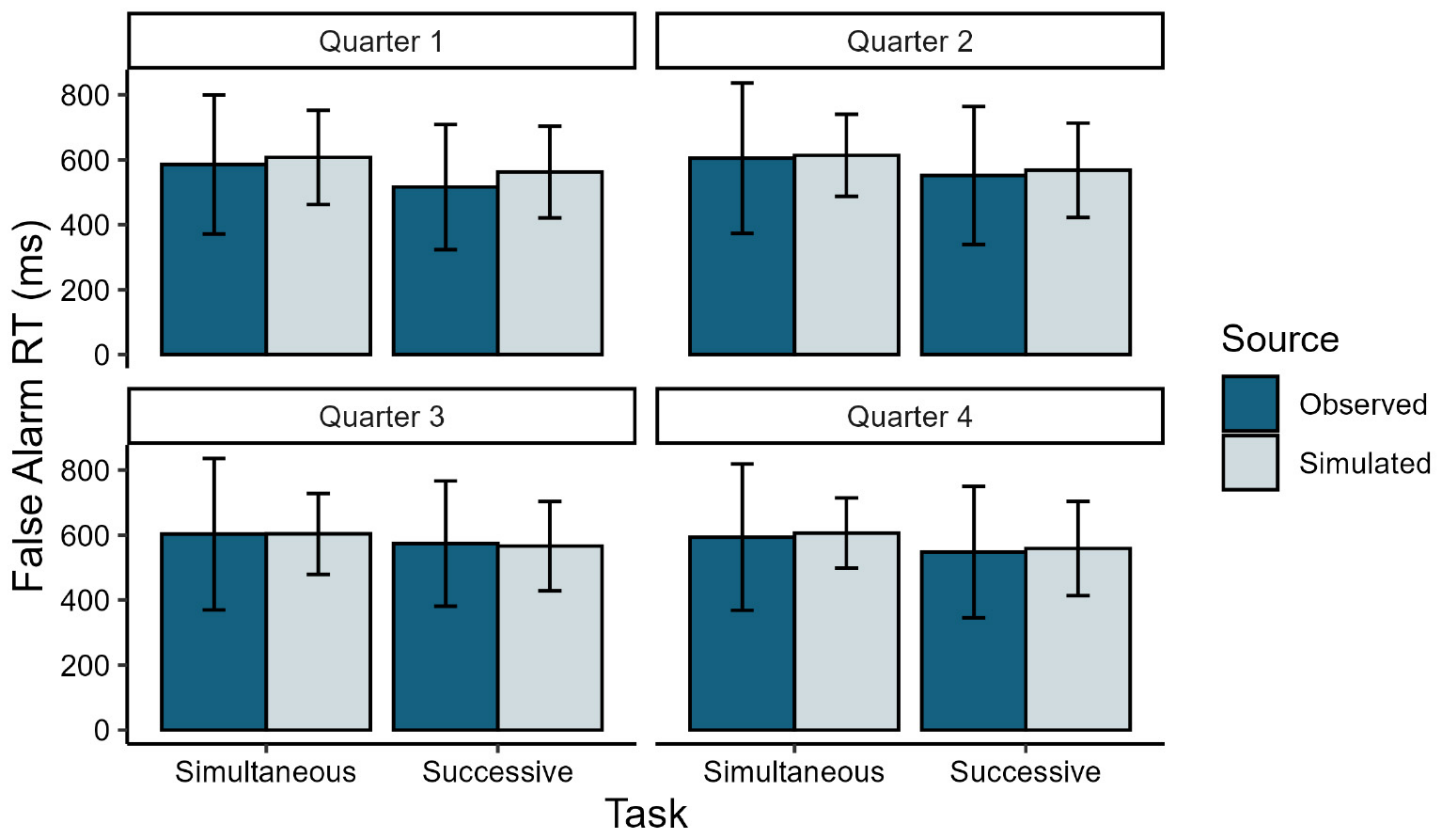
Reaction times for false alarm responses across tasks for both observed and simulated data.

As depicted in Figure 3, the effects of Task, *F*_(1, 103)_ = 1.96, *p* = 0.17, $\eta_{p}^{2}$ = 0.02, and Quarter, *F*_(3, 103)_ = 0.73, *p* = 0.54, $\eta_{p}^{2}$ = 0.02, were nonsignificant, as was the interaction of the factors, *F*(3,103) = 1.03, *p* = 0.38, $\eta_{p}^{2}$ = 0.03. Given our hypotheses, we examined contrasts for interaction of Task and Quarter. There was no difference between the Simultaneous and Successive tasks in Quarter 1 [Simultaneous, *M* = 640, *SD* = 142; Successive, *M* = 550, *SD* = 94; *t*(22.63) = 2.02, *p* = 0.06], Quarter 2 (Simultaneous, *M* = 653, *SD* = 182; Successive, *M* = 603, *SD* = 130; *t*(21.35) = 0.83, *p* = 0.41), Quarter 3 (Simultaneous, *M* = 672, *SD* = 177; Successive, *M* = 610, *SD* = 110; *t*(19.50) = 1.09, *p* = 0.29), or Quarter 4 [Simultaneous, *M* = 570, *SD* = 192; Successive, *M* = 615, *SD* = 153, *t*_(18.57)_ = −0.64, *p* = 0.53].

### 3.2 Electrocortical

#### 3.2.1 ERN peak amplitude

**Figure 4:**
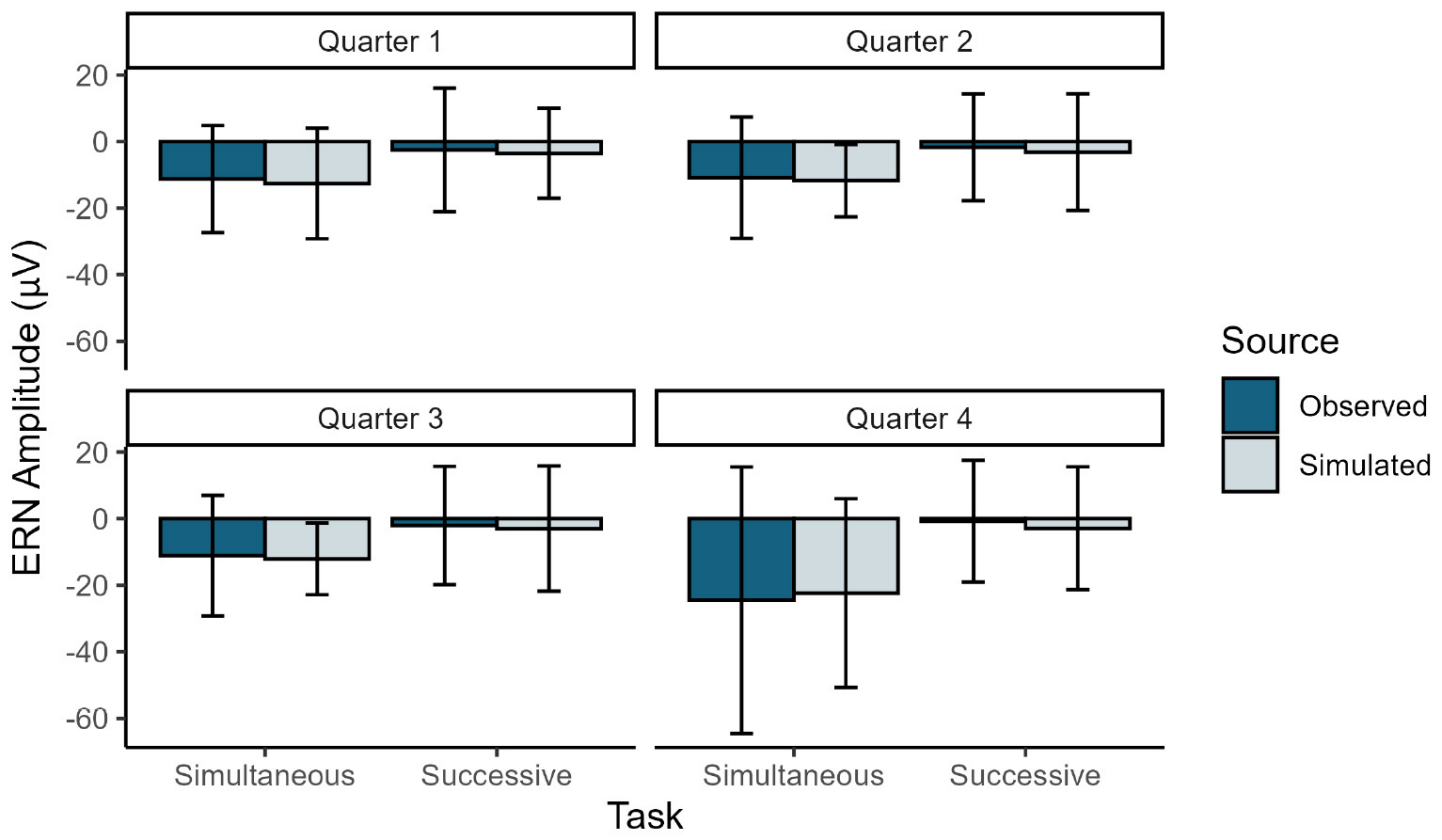
Peak amplitudes for observed and simulated ERNs across conditions and quarters.

As illustrated in Figure 4, ERN peak amplitude in the experimental task differed significantly as a function of Task, *F*_(1, 103)_ = 25.47, *p* < 0.001, $\eta_{p}^{2}$ = 0.20, though the effect of Quarter, *F*(3,103) = 0.37, *p* = 0.77, $\eta_{p}^{2}$ = 0.01, and the interaction of Task and Quarter, *F*_(3, 103)_ = 0.61, *p* = 0.61, $\eta_{p}^{2}$ = 0.02, were nonsignificant. Again, given our hypotheses, we examined the interaction of Task and Quarter, observing a significant difference in Quarter 1 between Simultaneous (*M* = −11.22, *SD* = 9.33) and Successive (*M* = 0.31, *SD* = 9.96), *t*_(27)_ = −3.22, *p* = 0.003; in Quarter 2, Simultaneous (*M* = −14.49, *SD* = 8.44) and Successive (*M* = −0.47, *SD* = 4.61), *t*_(27)_ = −5.34, *p* < 0.001; in Quarter 3, Simultaneous (*M* = −10.60, *SD* = 9.99) and Successive (*M* = −1.63, *SD* = 11.02), *t*_(25.93)_ = −2.26, *p* = 0.03, though this was nonsignificant in Quarter 4, Simultaneous (*M* = −18.56, *SD* = 30.44) and Successive (*M* = 0.27, *SD* = 16.71), *t*_(14.41)_ = −1.86, *p* = 0.08.

#### 3.2.2 ERN peak latency

ERN peak latency did not differ for Task, *F*_(1, 103)_ = 0.28, *p* = 0.60, $\eta_{p}^{2}$ < 0.01, nor Quarter, *F*_(3, 103)_ = 0.26, *p* = 0.85, $\eta_{p}^{2}$ = 0.01, nor the interaction of these, *F*_(3, 103)_ = 1.40, *p* = 0.25, $\eta_{p}^{2}$ = 0.04. Based on our *a priori* hypotheses, we examined the interaction, observing no Quarter 1 difference between Simultaneous (*M* = 63.60 msec, *SD* = 29.75) and Successive (*M* = 78.79, *SD* = 9.09), *t*_(15.26)_ = −1.83, *p* = 0.09, in Quarter 2 (Simultaneous *M* = 81.07, *SD* = 32.20; Successive, *M* = 69.24, *SD* = 20.26, *t*_(19.66)_ = 1.14, *p* = 0.27), in Quarter 3 [Simultaneous *M* = 40.26, *SD* = 11.17; Successive, *M* = 24.87, *SD* = 6.42, *t*_(19.43)_ = 1.14, *p* = 0.41], or in Quarter 4 [Simultaneous *M* = 27.68, *SD* = 8.35; Successive, *M* = 30.37, *SD* = 7.84, *t*_(22.77)_ = 0.25, *p* = 0.80].

### 3.3 Interim discussion

The accuracy and response time patterns reflected a canonical vigilance decrement: Reaction times became slower across blocks of trials while accuracy decreased, particularly between Quarters 1 and 2. Contrary to our hypotheses, however, there were no significant group differences in overall response patterns. Thus, in the aggregate, participants performed similarly on successive and simultaneous discrimination trials.

More focused analyses of the false alarm data, however, do highlight condition and time-on-task differences that align with our *a priori* hypotheses. Specifically, participants showed significantly higher false alarm rates during simultaneous discrimination tasks, where false alarm errors increased between the first and second blocks and decreased slightly between the last two blocks. Conversely, false alarm rates during successive discrimination decreased between the first two blocks of trials and increased slightly across the remaining 2 quarters. Despite these differences, false alarm latency patterns were not significantly different between experimental conditions, nor did they significantly change across the vigil.

Analyses of EEG patterns during false alarm errors also confirm our hypotheses: ERN peak amplitudes during simultaneous discrimination were significantly greater in magnitude than those during successive discrimination. Additionally, ERN amplitude during simultaneous discrimination increased in magnitude and variance across time-on-task, although this pattern was not statistically significant due to high variance. In contrast, the average ERN peak amplitude for false alarm errors during successive discrimination was small in magnitude and did not change across the task. The average peak latency of these ERNs did not differ significantly between conditions or across blocks of trials.

The results of the experiment only partially met our expectations. During simultaneous discrimination, false alarm rates and ERN amplitudes predictably increased across time-on-task, reflecting decreases in attentional resources (e.g., Thomson et al., 2015) and increases in effortful performance monitoring (e.g., Clayson et al., 2025). As expected, ERN amplitudes during successive discrimination were small and did not change across the vigil, reflecting an attenuation in conflict monitoring due to cognitive resources being allocated to working memory engagement; however, false alarm rates during successive discrimination were significantly lower than those for simultaneous discrimination, contrary to our predictions and prior research using similar tasks (Caggiano and Parasuraman, 2004; Helton and Russell, 2011).

The behavioral results can be partially explained by studies that found no vigilance decrements in tasks that use executive functions (e.g., Mart́ınez-Pérez et al., 2023; Zholdassova et al., 2021); however, the near-zero ERN amplitudes for successive discrimination trials are unexpected. An alternative hypothesis is that the active maintenance of a template in working memory forces participants to continuously maintain executive resources (e.g., Luna et al., 2022), reducing the cognitive effort that is typically expended during speeded Go/No-go responses and leading to fewer error responses. In this case, high-level cognitive monitoring would be spread across the trial and not localized to the small window of time encasing the presentation of the stimulus, the response, and the period immediately following the response, thus reducing the peak magnitude of an ERN following a false alarm response.

## 4 Modeling formalism

We investigated the potential influence of continued exertion of cognitive control by simulating both the behavioral and electrophysiological outcomes of the study. Specifically, we expressed parallel distributed processing accounts of ACC functioning (Botvinick et al., 2001; Yeung et al., 2004) and expected value of cognitive control (Botvinick and Cohen, 2014; Shenhav et al., 2013) using models of both the simultaneous and successive discrimination tasks.

### 4.1 Processing framework

**Figure 5:**
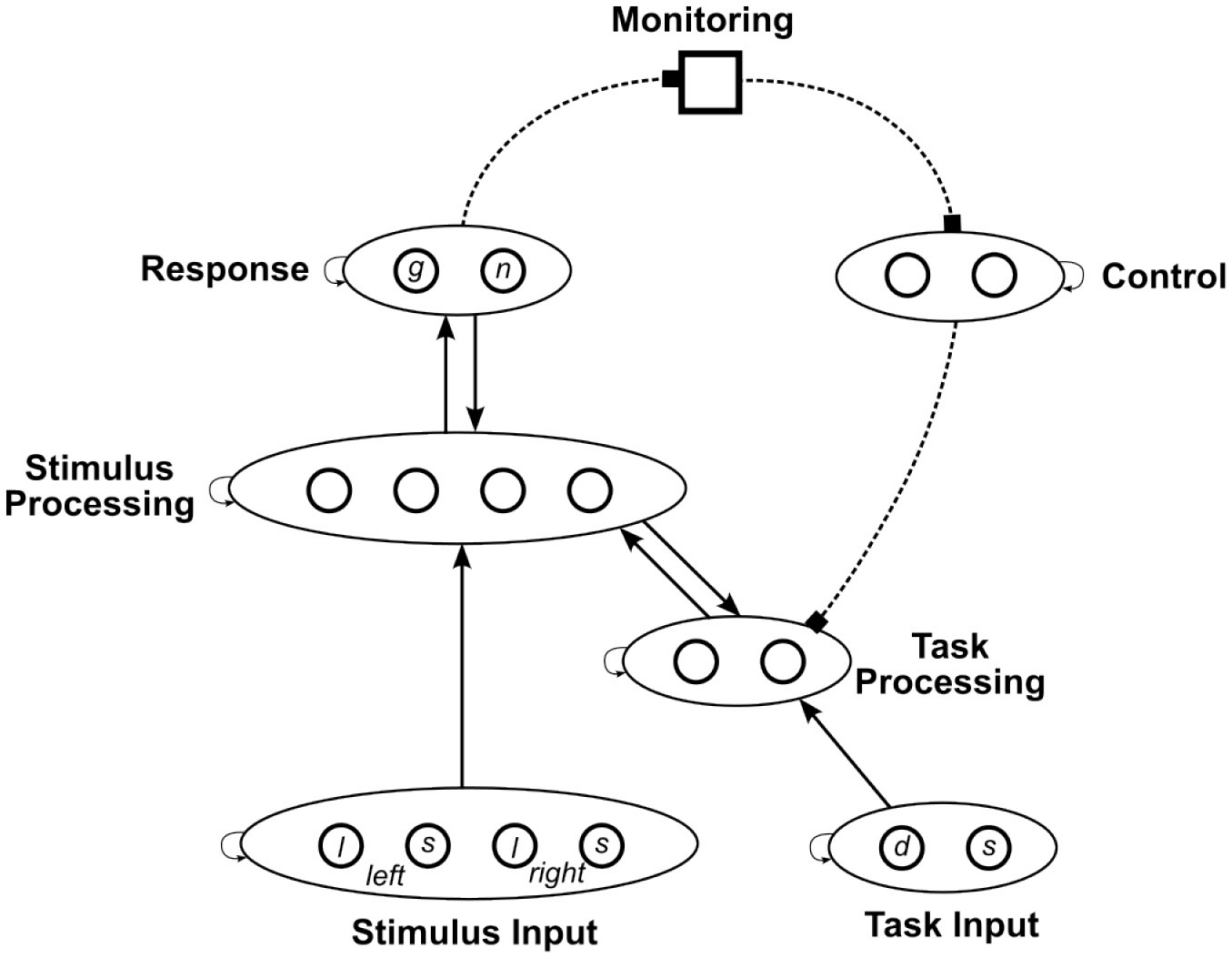
Simultaneous task model.

The flow of information in the proposed model follows a relatively simple pathway, particularly in the case of trials during the simultaneous discrimination condition (Figure 5). Stimuli, or input to the model, are represented as two objects (*left* and *right* lines) that are either short (*s*) or long (*l*), resulting in 4 nodes that are either “on” (1) or “off” (0). This information is then processed by a hidden Stimulus Processing layer that determines whether the lines are of similar or different lengths. The Stimulus Processing layer is also informed by signals pertaining to task instructions, i.e., whether to respond if the 2 lines are different (*d*) or the same (*s*). Task information is integrated with stimulus information after it passes through an intermediary, hidden layer (Task Processing). The model then makes a determination on whether to provide (“Go”; *g*) or withhold (“No go”; *n*) a response based on the combined stimulus and task activations.

**Figure 6:**
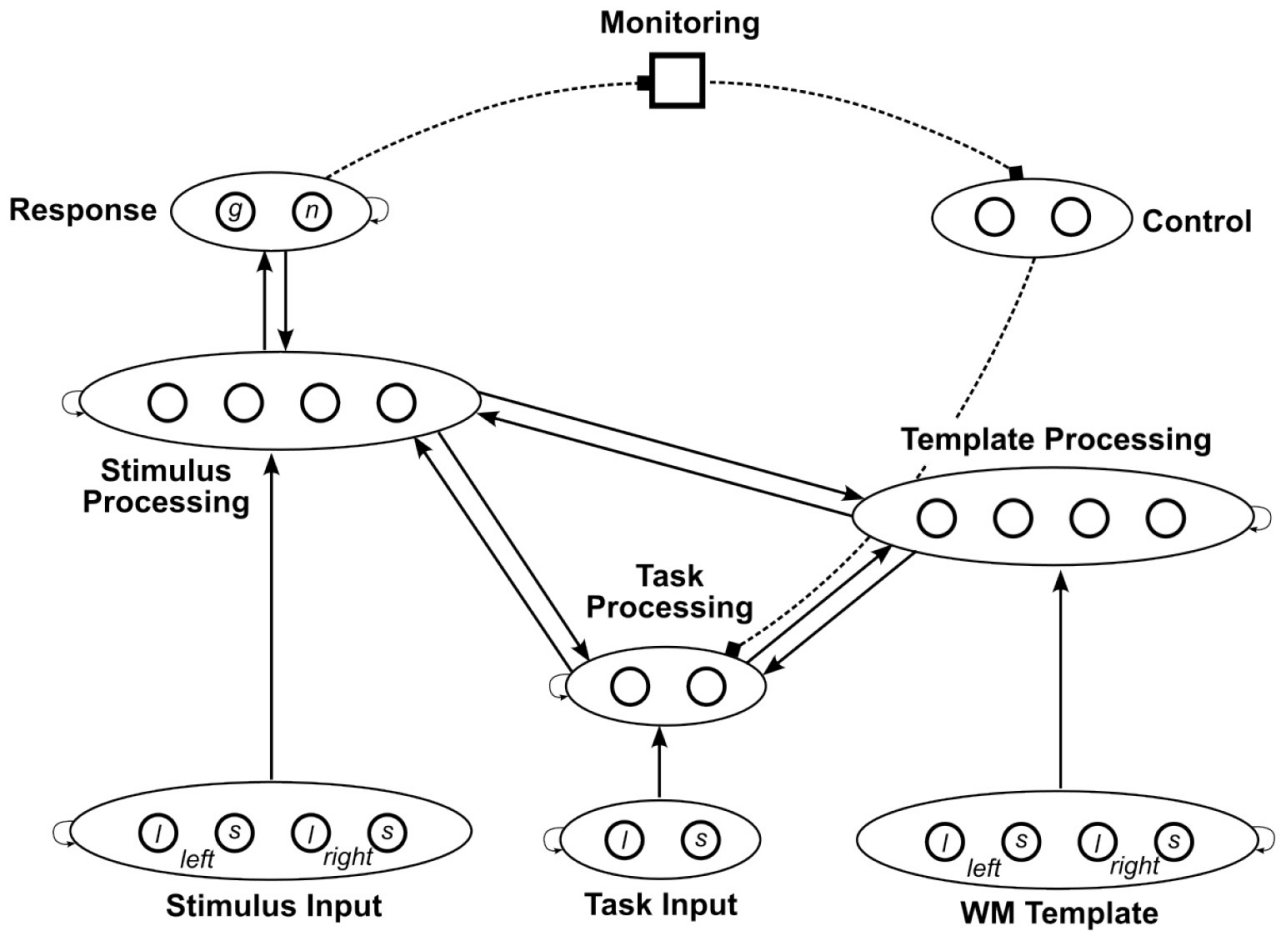
Successive task model.

In the successive discrimination task model (Figure 6), the structure, though similar to the simultaneous task, is compounded by a match-to-sample process that is needed to compare presented stimuli to a target set held in memory (e.g., Hyun et al., 2009). During such trials, a parallel information stream that includes the target stimulus template is activated and connected to a separate Template Processing layer, but only to classify the target pattern as being either both long or both short. The Stimulus Processing layer adjudicates whether the trial stimulus and target configuration match and prompts either a “Go” (*g*) or “No-go” (*n*) response. The activations of the units in the Response layer, in turn, determine the probability and latency of model responses to the experimental task. The Task nodes project onto the Stimulus and Template layers and help the model whether to respond to stimuli with two short lines (*s*) or two long lines (*l*).

Both models include a feedback loop that monitors the activations of the Response units and then projects a control signal back to the Task Processing layer. This signal is responsive to the level of conflict between the two Response nodes and either excites further activity (low conflict) or dampens subsequent responses (high conflict). Importantly, this feedback loop serves different purposes in the two tasks: In the simultaneous task model, continuous control is exerted on a processing node that monitors direct stimulus processing. In the successive task model, however, control also governs the sustained representation of the target template in working memory. The exact mechanisms of this feedback loop are further explained in the next section (Section 4.2).

### 4.2 Computing framework

We constructed the model in Python using the PsyNeuLink package (*PsyNeuLink*, https://princetonuniversity.github.io/PsyNeuLink)^1^ with governing mechanics similar to those used by Cohen, Botvinick, and colleagues (Botvinick et al., 2001; Botvinick and Cohen, 2014; Cohen and Huston, 1994; Yeung et al., 2004) and the expected value of control cognitive modeling formalism (Shenhav et al., 2013). In both the simultaneous (Figure 5) and successive (Figure 6) task models, pathways between inputs (i.e., stimulus and task information) and processing layers are unidirectional while those between processing layers and to the response layers are bidirectional and excitatory.

Values are propagated across layers in a manner similar to standard neural network architectures: Unit activations in a receiving layer are a function of the unit activations for a sending layer (net input), weight values between the two layers, and a monotonic activation function that constrains the activity of the units in the receiving layer. The current model uses a standard logistic activation function, 1/1+*e*^−*kx*^, where *x* is the net input and *k* is a gain parameter that controls the slope of the function.

**Figure 7:**
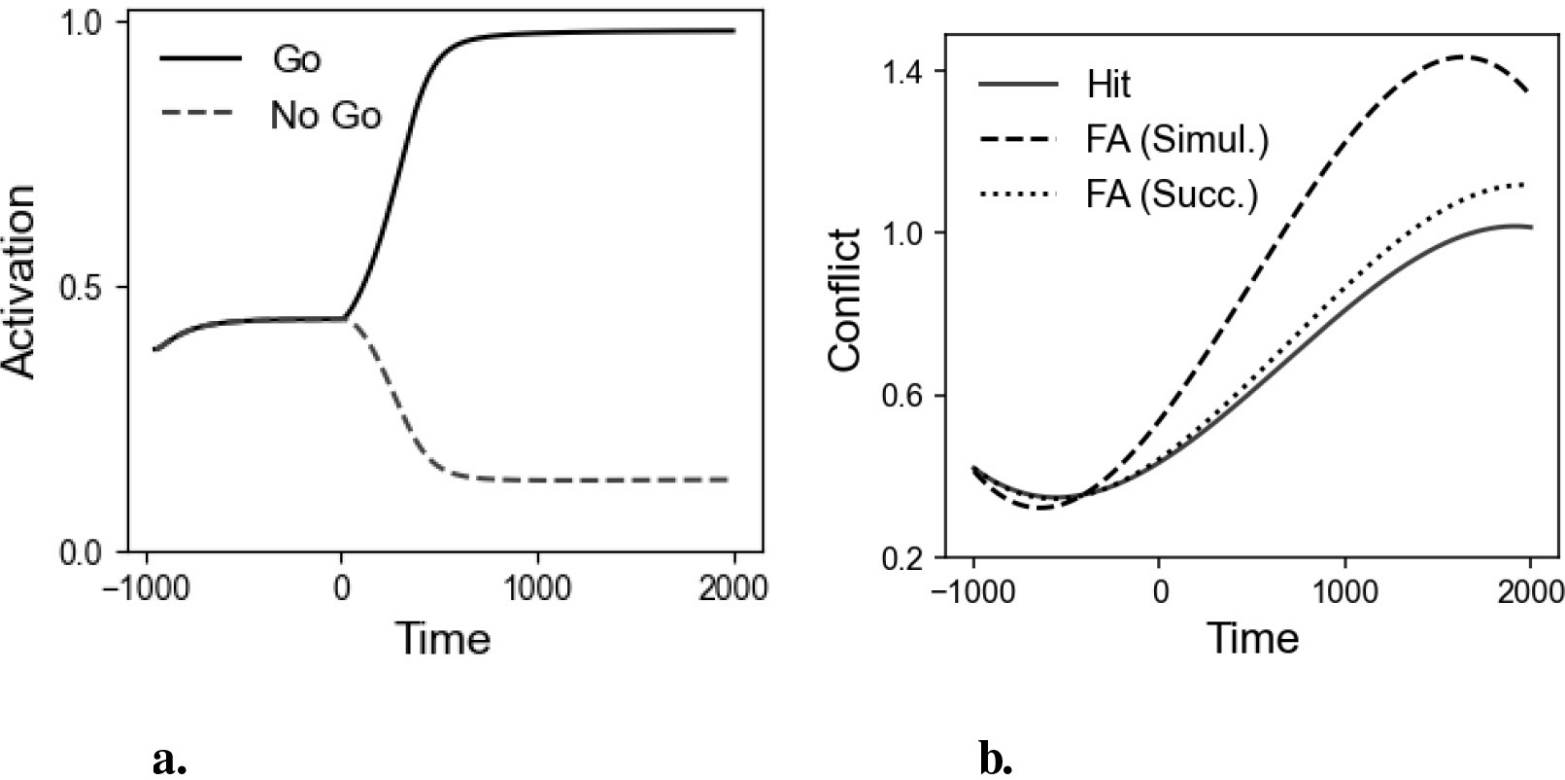
Illustrations of simulated response node activations **(a)** and response conflict **(b)** across cycles. ACC activation is simulated by taking the difference in simulated conflict between hit and false alarm trials.

At the beginning of each trial, the model incurs an initial “settling” period in which Task, but not Stimulus, inputs are processed by the model over a short period, e.g., 1,300 − 1,700 cycles (“milliseconds”). This helps to prime the model to the task and simulate the period of time before stimulus onset, i.e., the variable interstimulus interval (ISI). Afterwards, the model processes both the Task and Stimulus Inputs for 150 cycles and continues for a variable duration, either until a decision threshold is exceeded and the model produces a response (“Go”) or until the maximum trial duration is reached (“No-go”). Similar to computational accounts of evidence accumulation (e.g., Ratcliff, 1978; Usher and McClelland, 2001), the model compares the activation values of the two Response nodes to a threshold value, and the node whose activation reaches the value first is considered the “winner”, or chosen option (e.g., Figure 7a). For “Go” responses, the trial is terminated immediately after the *g* node's activation exceeds the threshold, and the number of cycles it took to reach the threshold is considered to be the response latency. For “No-go” responses, however, the trial continues until it reaches the maximum duration, even after the *n* node's activation exceeds the response threshold. Thus, the model either withholds or provides an overt response on the basis of the simulated activations of the *n* and *g* Response nodes, respectively.

Importantly, the two Response nodes share a mutual connection that dictates levels of inhibition between the two types of responses (e.g., Figure 7b). The interactions between node activations and inhibitory activity reflect overall conflict in responding (e.g., Botvinick et al., 2001; Yeung et al., 2004): When one node is highly active and the other dampened, there is a low level of conflict. When both nodes are activated simultaneously, however, the level of conflict remains high until either the competition has been resolved or until the next trial begins. Similar to Botvinick et al. (2001), conflict (or energy) is measured as the Hopfield (1982) energy in a recurrent network, i.e.,:


$$\begin{array}{l} \text{Conflict}=-\sum \sum a_{i}a_{j}w_{i,j}, \end{array}$$ (1)

which measures the product of competing unit activations *a* and the inhibitory weights *w* between them. The energy observed from this metric provides a simulation of ACC activation that reflects conflict and response monitoring, such as the ERN. Like other response conflict models (e.g., Yeung et al., 2004), we simulated ERNs as the difference in response Conflict between hits and false alarms.

This conflict signal is propagated as a recurrent signal back to the Task Processing layer through nodes that control task performance, specifically projecting to nodes that reflect the target response, i.e., the presence of “similar” (*s*) or “different” (*d*) line lengths in the simultaneous task or the “both short” (*s*) and “both long” (*l*) line lengths in the successive task. While other researchers have used more complex formulations for integrating the control signal (e.g., Botvinick et al., 2001), we combine the control signal with Task Processing via a scalar product. Thus, the Control layer provides a top-down signal reflecting task demands, similar to the role of frontal cortical regions in executive tasks (e.g., ACC; Shenhav et al., 2013): Positive signals enhance performance on the simultaneous and successive tasks while negative signals dampen performance. In particular, the values that project from the Control layer are used to modulate the growth rate of the logistic activation function in the Task Processing layer (e.g., Botvinick et al., 2001), such that higher levels of Conflict lead to smaller task activations and, in turn, more “controlled” responses.

In our model, the Control nodes are governed by two separate signals: conflict (Equation 1) and effort. Whereas conflict acts as an inhibitory signal, effort is largely excitatory and can help innervate task performance, reflecting motivated on-task performance. The Control nodes, in turn, govern Stimulus Process in both task models as well as Working Memory Template Processing in the successive task model. In previous computational frameworks, trade-offs between bottom-up activation and top-down control are integrated with parameters using power spectral density (PSD) values calculated after the experiment (Curley et al., 2024). In the current model, however, both neural and behavioral data are generated during the course of the simulation, providing an on-line account of effortful control.

### 4.3 Modeling results

**Figure 8:**
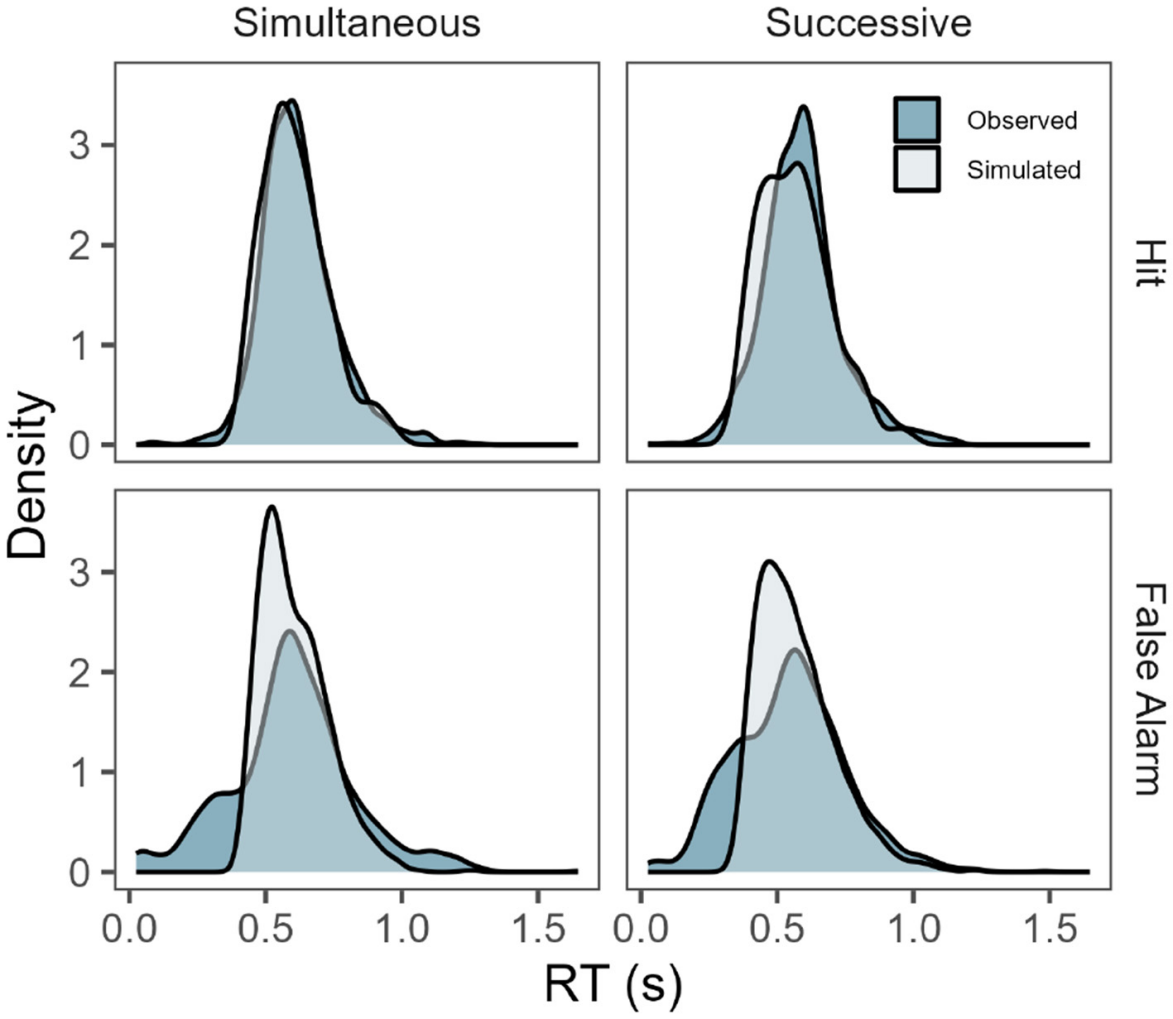
Comparisons of simulated and observed reaction time distributions across response conditions for hit and false alarm trials.

Overall, the task models faithfully simulated data patterns observed in the experiment. Specifically, a Mann-Whitney U Test indicated that the RTs simulated in the model for the Simultaneous, *z* = 0.42, *p* = 0.67, and Successive, *z* = 1.02, *p* = 0.31, tasks were not significantly different from the RTs observed in the study (Figure 8). A similar test on false alarm reaction times also failed to yield significant differences between the observed and simulated RTs for either the Simultaneous, *z* = 0.36, *p* = 0.72, or Successive, *z* = 1.22, *p* = 0.23, tasks.

Importantly, the ERNs generated by the computational models were similar to those observed in the results of the experiment. Specifically, the simulated peak amplitude values in the Simultaneous, *z* = 1.01, *p* = 0.31, and Successive, *z* = 0.81, *p* = 0.42, tasks were not significantly different from the values observed during the experiment, nor were the simulated peak latency values in the Simultaneous, *z* = 0.82, *p* = 0.43, and Successive, *z* = 0.99, *p* = 0.29, tasks. Thus, the addition of working memory template processing in the Successive take model replicated the differences in response, latency, and ERN patterns described in the Experimental Results (Section 3).

## 5 Discussion

The current study examined behavioral performance and the ERN during multi-block Go/No-go tasks. In particular, we compared behavior and neural metrics related to response errors between simultaneous and successive discrimination tasks. We predicted that ERN amplitude would decrease across trial blocks (c.f., Clayson et al., 2025), with attenuated ERN amplitudes during successive discrimination, reflecting higher levels of cognitive control emanating from working memory engagement (c.f., Caggiano and Parasuraman, 2004; Helton and Russell, 2011). The results of the experiment reflected significant differences in false alarm rates across time-on-task between the two conditions. Interestingly, we found no significant differences between tasks or across blocks for false alarm reaction times or ERN latencies, but significantly higher ERN amplitudes for error responses during simultaneous discrimination, particularly during the last quarter. Neurocognitive models of the tasks based on parallel distributed processing (PDP) accounts of the ERN (e.g., Botvinick et al., 2001; Cohen and Huston, 1994) replicate the results observed during the experiment.

Many aspects of these results replicated several key patterns observed in prior vigilant attention tasks (c.f., Thomson et al., 2016): response accuracy decreased slightly while response latency increased, particularly after the first block of trials following practice (Quarter 1). Other results departed from prior studies; specifically, reaction times did not significantly differ between the simultaneous and successive discrimination tasks, despite the higher working memory demands in the latter (c.f., Caggiano and Parasuraman, 2004). Observed false alarm rates were significantly greater during successive discrimination, contrary to our expectations based on prior research on the role of working memory in the vigilance decrement. Additionally, ERN amplitudes for false alarm errors were stable across trials and were numerically greater during simultaneous discrimination compared to successive discrimination, particularly in the last block of trials (Quarter 4), where the difference was statistically significant. This is in opposition to prior research indicating that ERN amplitudes decrease across time-on-task (Clayson et al., 2025; Xiao et al., 2015).

An interesting replication was that from Study 2 of Clayson et al. (2025), where ERN amplitudes generally decreased across time (replicating results from Clayson et al., 2012; Moore et al., 2017, among others), with the exception of those associated with the Go/No-go task, which increased in amplitude and variability toward the end of the vigil. This is similar to the between-task differences in the current study, where ERN amplitudes were significantly greater in Quarter 4 trials during simultaneous discrimination. These differences in error-related waveforms between the simultaneous and successive discrimination tasks were expected given the additional working memory demands of the successive task. The simultaneous discrimination task and the one used by Clayson et al. (2025) only require discrimination of simple visual stimuli, i.e., vertical line similarity in the former and triangle orientation in the latter. Successive discrimination, however, requires a comparison to a target template that must be held in working memory throughout the trial block and, in turn, a different type of sustained engagement (c.f., Caggiano and Parasuraman, 2004; Helton and Russell, 2011).

The main question of the current study is why there are differences in the ERN waveform following false alarms between conditions and how they relate to sustained effort and control. The results of the current study suggest that these differences reflect relationships between task demands, changes in effort allocation across time-on-task, and when control and monitoring mechanisms are allocated during the course of experimental trials. Similar to Clayson et al. (2025), we interpret overall differences in ERN amplitudes between tasks as reflecting differences in the allocation of effort before and after a response is given: During successive discrimination, pre-stimulus cognitive effort is required for the maintenance of a target template in working memory for comparison to the presented stimulus. During simultaneous discrimination, however, this pre-stimulus effort is not required, placing a higher degree of control and error monitoring needed to inhibit a prepotent response. These increases in monitoring and control during simultaneous discrimination are not always enough to offset decrements due to time-on-task, however, which can lead to increases in ERN amplitude without subsequent decreases in false alarm rates and latency. This argument is similar to several lines of research examining the locus of control in common response inhibition tasks (e.g., MacLeod et al., 2003; Paap et al., 2020; Stahl et al., 2014).

Parallel distributed processing models simulating both behavioral performance and ERN activity provide evidence in favor of processing differences between the two experimental tasks. In the simultaneous discrimination model (Figure 5), Stimulus Input and Stimulus Processing layers are directly connected to the two Response nodes, and this pathway is only modulated by the Task Processing layer. Thus, the presented stimulus provides a strong projection to a final response and requires a high degree of feedback (via the Conflict signal; see Equation 1 and Figure 7b) following error processing to override a prepotent response during critical events compared to non-critical events. In the successive discrimination model (Figure 6), however, the projections to the Response layer are additionally adjudicated by a Template Processing layer that helps determine the match between the presented stimulus and the one held in working memory (via the WM Template layer). Further examination of the Conflict signal —the basis of the simulated ERNs —and Stimulus Processing layer activity indicates that the addition of the Template layers in the successive task model reduces node activity in the Stimulus Processing layer that gives rise to competition in the Response layer.

An interesting finding in the current study were the increases in magnitude and variance in ERN amplitudes during the last block of trials (Quarter 4) during simultaneous discrimination, though only trending toward significance. This indicates that a) higher degrees of feedback and control following response errors are allocated after longer task durations and b) ERNs toward the end of a vigil show variability between and within individuals. This is generally consistent with a large number of studies showing increases in error rates across time-on-task (e.g., Gunzelmann et al., 2009; See et al., 1995; Warm et al., 2018), but also reflects an emerging body of literature touting the role of effortful compensation toward the end of a vigil (e.g., Borghetti et al., 2024; Curley et al., 2024; Morris et al., 2023). In particular, the current study extends hybrid frameworks of fatigue and compensatory control (Borghetti et al., 2024; Curley et al., 2024; Veksler and Gunzelmann, 2018) by explicating the role of motivation and control via computational cognitive modeling. An important contribution of the current study to these theories is the direct investigation of control behavior across time-on-task following response errors, particularly for experimental tasks that differ only with respect to reliance on working memory. Future studies might help improve this hybrid approach by examining factors that will moderate these control behaviors, such as changes to the occurrence of target (valid) trials and individual differences in cognitive ability.

## 6 Conclusions

In summary, the present study examined indices of error monitoring and control behavior in two sustained visual discrimination tasks using traditional experimentation and novel modeling techniques. While false alarm rates were not different between tasks, ERN amplitudes were significantly greater for the simultaneous discrimination task compared to the successive discrimination task—a difference that increased at the end of the vigil. Neurocognitive models of the tasks replicated these results with a particular focus on conflict signals emanating from inhibitory activity at response, which formed the basis of simulated ERNs. These findings underscore the role of sustained effortful control in response error monitoring and advantages of hybrid approaches to investigating fatigue.

The results of the experiment and computational models provide context for the role of executive resource allocation in theories of the vigilance decrement. Specifically, the maintenance of a target configuration during sustained visual discrimination tasks can require the effortful allocation of resources both pre- and post-stimulus and help alleviate the high response demands needed in most vigilance tasks (Luna et al., 2022), leading to lower false alarm rates and conflict processing during false alarm errors. Further research will be needed to understand why the vigilance decrement was decreased during successive vs. simultaneous discrimination (instead of the opposite; c.f., Caggiano and Parasuraman, 2004; Helton and Russell, 2011; Warm and Dember, 1998) and to further understand how the allocation of executive resources within and between experimental trials can help reduce vigilance decrements.

## Data Availability

The original contributions presented in the study are included in the article/supplementary material, further inquiries can be directed to the corresponding author.

## Footnotes

1 Code for the Successive and Simultaneous models is publicly available and can be found at the following link: https://osf.io/a89fs.
